# Supplementary material for: Impact of decreasing the proportion of higher energy foods and reducing portion sizes on food purchased in worksite cafeterias: A stepped-wedge randomised controlled trial
Source: PLoS Med. 2021 Sep 14;18(9):e1003743. doi: 10.1371/journal.pmed.1003743 (PMC8439477; doi:10.1371/journal.pmed.1003743)
Supplement: S1 Text — Table A: Criteria for categorising higher and lower energy foods. Table B: Energy purchased per day from intervention categories: full models. Table C: Energy purchased per day from nonintervention categories: full models. Table D: Energy purchased per day from all categories: full models. Table E: Revenue per day from all categories: full models. Table F: A comparison of the primary analysis and sensitivity analysis for the primary outcome. Table G: Table of correlations between intervention implementation and effectiveness. (DOCX) [file pmed.1003743.s001.docx]

**Supplement**

| Categories | Items | Cut-off for higher energy | Higher energy | Lower energy | Target proportion of higher energy products during Availability |
| --- | --- | --- | --- | --- | --- |
| Cooked main meals (excluding breakfast) | Complete main meals | 530+ kcals | e.g., roast leg of pork, stuffing, apple sauce, roast potatoes & carrots | e.g., smoked haddock kedgeree with chef’s salad; broccoli & stilton quiche with chef’s salad | 50% |
|  | Main meal without a side | 330+ kcals | e.g., battered fillet of pollock; BBQ slaw chicken burger | e.g., grilled salmon | 50% |
|  | Sides | 10%+ fat | e.g., chips; roast potatoes; buttered noodles | e.g., boiled potatoes; basmati rice | Only 1 higher energy side |
| Snacks | Savoury snacks | 120+ kcals | e.g., full-fat thick cut crisps | e.g., popped crisps; selected popcorns | 75% |
|  | Sweet snacks | 150+ kcals | e.g., standard chocolate bars; biscuits | e.g., selected cereal bars; rice cakes | 80% |
| Drinks | Cold drinks (100% fruit juice & smoothies excluded) | 50+ kcals | e.g., full sugar soda; juice-based drinks with added sugar; energy drinks | e.g., water; diet soda; diet energy drinks | 50% |
| Dessert | Dessert pots, hot desserts | 310+ kcals | e.g., chocolate sundae; lemon sponge | e.g., jelly; French apple and pear tart | 50% |
| Bakery |  | 513 kcals (median split) | e.g., classic Victoria sponge | e.g., marmalade cake | 50% |

**Table A.** Criteria for categorising higher and lower energy foods

*Note.* The decision regarding which products to remove was a negotiation between the catering providers (who wanted to keep certain high selling products) and the research team (who wanted to remove the highest energy products). We were successful in making the case for replacing the highest energy main meals (i.e. pizza). It did not prove possible to replace the highest energy side dish (i.e. chips). It was, however, possible to reduce the portion size of the chips and other higher energy sides such as roast potatoes and wedges.

**Full models**

**Table B.** Energy purchased per day from intervention categories: full models

|  | Reference: Baseline | | | | Reference: Availability | | | |
| --- | --- | --- | --- | --- | --- | --- | --- | --- |
|  | Estimate | SE | *t* value | *p* | Estimate | SE | *t* value | *p* |
| Modelling of the mean (identity link): |  |  |  |  |  |  |  |  |
| Intercept | 14969.5 | 1722.24 | 8.69 | < 0.001 | 9969.6 | 2411.79 | 4.13 | < 0.001 |
| Transactions | 319.7 | 4.49 | 71.20 | < 0.001 | 319.7 | 3.09 | 103.60 | < 0.001 |
| Temperature (°C) | 36.8 | 63.84 | 0.58 | 0.565 | 36.8 | 89.34 | 0.41 | 0.681 |
| Time (Days; Baseline) | -1841.4 | 1605.49 | -1.15 | 0.252 | -1841.4 | 1927.38 | -0.96 | 0.339 |
| Time (Days; Availability) | -174.3 | 467.23 | -0.37 | 0.709 | -174.3 | 1905.58 | -0.09 | 0.927 |
| Time (Days; Availability plus Size) | -157.4 | 244.14 | -0.65 | 0.519 | -157.4 | 2148.52 | -0.07 | 0.942 |
| Baseline | *reference* | *reference* | *reference* | *reference* | 4999.9 | 1630.93 | 3.07 | 0.002 |
| Availability | -4999.9 | 1146.96 | -4.36 | < 0.001 | *reference* | *reference* | *reference* | *reference* |
| Availability plus Size | -11925.9 | 1157.48 | -10.30 | < 0.001 | -6926.0 | 1552.67 | -4.46 | < 0.001 |
|  |  |  |  |  |  |  |  |  |
| Modelling of the variance (log link): |  |  |  |  |  |  |  |  |
| Intercept | 10.0 | 0.02 | 472.29 | < 0.001 | 10.0 | 0.05 | 187.64 | < 0.001 |
| Site 2 | -0.2 | 0.06 | -3.90 | < 0.001 | -0.2 | 0.08 | -3.14 | 0.002 |
| Site 3 | 0.0 | 0.03 | 0.90 | 0.370 | 0.0 | 0.08 | 0.32 | 0.753 |
| Site 4 | -0.6 | 0.06 | -10.46 | < 0.001 | -0.6 | 0.08 | -8.23 | < 0.001 |
| Site 5 | 0.2 | 0.06 | 2.55 | 0.011 | 0.2 | 0.08 | 1.94 | 0.053 |
| Site 6 | 0.2 | 0.06 | 2.88 | 0.004 | 0.2 | 0.08 | 2.23 | 0.026 |
| Site 7 | 0.0 | 0.03 | 0.69 | 0.493 | 0.0 | 0.08 | 0.27 | 0.785 |
| Site 8 | -0.2 | 0.06 | -3.08 | 0.002 | -0.2 | 0.08 | -2.27 | 0.023 |
| Site 9 | -0.8 | 0.06 | -14.59 | < 0.001 | -0.8 | 0.08 | -11.05 | < 0.001 |
| Site 10 | 1.6 | 0.06 | 28.28 | < 0.001 | 1.6 | 0.08 | 21.73 | < 0.001 |
| Site 11 | -0.6 | 0.06 | -10.40 | < 0.001 | -0.6 | 0.08 | -8.22 | < 0.001 |
| Site 12 | -0.5 | 0.06 | -8.61 | < 0.001 | -0.5 | 0.08 | -6.86 | < 0.001 |
| Site 13 | 0.2 | 0.06 | 3.41 | 0.001 | 0.2 | 0.08 | 2.80 | 0.005 |
| Site 14 | 0.7 | 0.06 | 12.11 | < 0.001 | 0.7 | 0.08 | 9.21 | < 0.001 |
| Site 15 | 0.2 | 0.06 | 3.57 | < 0.001 | 0.2 | 0.08 | 2.91 | 0.004 |
| Site 16 | -0.6 | 0.06 | -10.85 | < 0.001 | -0.6 | 0.08 | -8.36 | < 0.001 |
| Site 17 | -0.1 | 0.05 | -2.05 | 0.041 | -0.1 | 0.08 | -1.39 | 0.165 |
| Site 18 | 0.2 | 0.07 | 3.12 | 0.002 | 0.2 | 0.08 | 2.67 | 0.008 |
| Site 19 | -0.4 | 0.06 | -6.08 | < 0.001 | -0.4 | 0.08 | -4.75 | < 0.001 |

**Table C.** Energy purchased per day from non-intervention categories: full models

|  | Reference: Baseline | | | | Reference: Availability | | | |
| --- | --- | --- | --- | --- | --- | --- | --- | --- |
|  | Estimate | SE | *t* value | *p* | Estimate | SE | *t* value | *p* |
| Modelling of the mean (identity link): |  |  |  |  |  |  |  |  |
| Intercept | 17843.0 | 1774.82 | 10.05 | < 0.001 | 9463.6 | 2239.41 | 4.23 | < 0.001 |
| Transactions | 299.4 | 2.46 | 121.88 | < 0.001 | 299.4 | 2.46 | 121.88 | < 0.001 |
| Temperature (°C) | -478.8 | 81.17 | -5.90 | < 0.001 | -478.8 | 81.17 | -5.90 | < 0.001 |
| Time (Days; Baseline) | -5836.6 | 1775.54 | -3.29 | 0.001 | -5836.6 | 1775.54 | -3.29 | 0.001 |
| Time (Days; Availability) | -405.2 | 1746.36 | -0.23 | 0.817 | -405.2 | 1746.36 | -0.23 | 0.817 |
| Time (Days; Availability plus Size) | 680.6 | 1946.62 | 0.35 | 0.727 | 680.6 | 1946.62 | 0.35 | 0.727 |
| Baseline | *reference* | *reference* | *reference* | *reference* | 8379.5 | 1511.94 | 5.54 | < 0.001 |
| Availability | -8379.5 | 1511.94 | -5.54 | < 0.001 | *reference* | *reference* | *reference* | *reference* |
| Availability plus Size | -8038.3 | 1419.26 | -5.66 | < 0.001 | 341.2 | 1406.07 | 0.24 | 0.808 |
|  |  |  |  |  |  |  |  |  |
| Modelling of the variance (log link): |  |  |  |  |  |  |  |  |
| Intercept | 9.7 | 0.05 | 181.02 | < 0.001 | 9.7 | 0.05 | 181.02 | < 0.001 |
| Site 2 | 0.4 | 0.08 | 4.96 | < 0.001 | 0.4 | 0.08 | 4.96 | < 0.001 |
| Site 3 | -0.1 | 0.08 | -1.64 | 0.102 | -0.1 | 0.08 | -1.64 | 0.102 |
| Site 4 | 0.3 | 0.08 | 3.74 | < 0.001 | 0.3 | 0.08 | 3.74 | < 0.001 |
| Site 5 | 0.0 | 0.08 | 0.46 | 0.649 | 0.0 | 0.08 | 0.46 | 0.649 |
| Site 6 | -0.2 | 0.08 | -2.17 | 0.030 | -0.2 | 0.08 | -2.17 | 0.030 |
| Site 7 | -0.1 | 0.08 | -1.13 | 0.259 | -0.1 | 0.08 | -1.13 | 0.259 |
| Site 8 | 0.2 | 0.08 | 2.51 | 0.012 | 0.2 | 0.08 | 2.51 | 0.012 |
| Site 9 | -0.7 | 0.08 | -9.54 | < 0.001 | -0.7 | 0.08 | -9.54 | < 0.001 |
| Site 10 | 0.7 | 0.08 | 8.77 | < 0.001 | 0.7 | 0.08 | 8.77 | < 0.001 |
| Site 11 | -0.0 | 0.08 | -0.07 | 0.944 | -0.0 | 0.08 | -0.07 | 0.944 |
| Site 12 | 0.0 | 0.08 | -0.01 | 0.989 | 0.0 | 0.08 | -0.01 | 0.989 |
| Site 13 | 0.5 | 0.08 | 6.06 | < 0.001 | 0.5 | 0.08 | 6.06 | < 0.001 |
| Site 14 | 0.5 | 0.08 | 7.04 | < 0.001 | 0.5 | 0.08 | 7.04 | < 0.001 |
| Site 15 | 0.7 | 0.08 | 8.90 | < 0.001 | 0.7 | 0.08 | 8.90 | < 0.001 |
| Site 16 | -0.0 | 0.08 | -0.13 | 0.897 | -0.0 | 0.08 | -0.13 | 0.897 |
| Site 17 | 0.3 | 0.08 | 4.29 | < 0.001 | 0.3 | 0.08 | 4.29 | < 0.001 |
| Site 18 | 0.2 | 0.08 | 1.99 | 0.046 | 0.2 | 0.08 | 1.99 | 0.046 |
| Site 19 | -0.2 | 0.08 | -2.38 | 0.017 | -0.2 | 0.08 | -2.38 | 0.017 |

**Table D.** Energy purchased per day from all categories: full models

|  | Reference: Baseline | | | | Reference: Availability | | | |
| --- | --- | --- | --- | --- | --- | --- | --- | --- |
|  | Estimate | SE | *t* value | *p* | Estimate | SE | *t* value | *p* |
| Modelling of the mean (identity link): |  |  |  |  |  |  |  |  |
| Intercept | 28907.1 | 2220.51 | 13.02 | < 0.001 | 20039.5 | 2757.56 | 7.27 | < 0.001 |
| Transactions | 612.6 | 3.42 | 179.15 | < 0.001 | 612.6 | 3.42 | 179.15 | < 0.001 |
| Temperature (°C) | -278.9 | 100.63 | -2.77 | 0.006 | -278.9 | 100.63 | -2.77 | 0.006 |
| Time (Days; Baseline) | -4069.7 | 2200.78 | -1.85 | 0.065 | -4069.7 | 2200.78 | -1.85 | 0.065 |
| Time (Days; Availability) | -2594.0 | 2163.05 | -1.20 | 0.231 | -2594.0 | 2163.05 | -1.20 | 0.231 |
| Time (Days; Availability plus Size) | 3046.8 | 2418.44 | 1.26 | 0.208 | 3046.8 | 2418.44 | 1.26 | 0.208 |
| Baseline | *reference* | *reference* | *reference* | *reference* | 8867.6 | 1865.92 | 4.75 | < 0.001 |
| Availability | -8867.6 | 1865.92 | -4.75 | < 0.001 | *reference* | *reference* | *reference* | *reference* |
| Availability plus Size | -16030.4 | 1765.06 | -9.08 | < 0.001 | -7162.8 | 1740.80 | -4.12 | < 0.001 |
|  |  |  |  |  |  |  |  |  |
| Modelling of the variance (log link): |  |  |  |  |  |  |  |  |
| Intercept | 9.8 | 0.05 | 183.96 | < 0.001 | 9.8 | 0.05 | 183.96 | < 0.001 |
| Site 2 | -0.0 | 0.08 | -0.59 | 0.553 | -0.0 | 0.08 | -0.59 | 0.553 |
| Site 3 | 0.5 | 0.08 | 5.73 | < 0.001 | 0.5 | 0.08 | 5.73 | < 0.001 |
| Site 4 | -0.1 | 0.08 | -1.06 | 0.289 | -0.1 | 0.08 | -1.06 | 0.289 |
| Site 5 | -0.2 | 0.08 | -2.25 | 0.024 | -0.2 | 0.08 | -2.25 | 0.024 |
| Site 6 | 0.5 | 0.08 | 6.49 | < 0.001 | 0.5 | 0.08 | 6.49 | < 0.001 |
| Site 7 | 0.2 | 0.08 | 2.44 | 0.015 | 0.2 | 0.08 | 2.44 | 0.015 |
| Site 8 | -0.0 | 0.08 | -0.33 | 0.742 | -0.0 | 0.08 | -0.33 | 0.742 |
| Site 9 | -0.6 | 0.08 | -7.22 | < 0.001 | -0.6 | 0.08 | -7.22 | < 0.001 |
| Site 10 | 2.0 | 0.08 | 26.18 | < 0.001 | 2.0 | 0.08 | 26.18 | < 0.001 |
| Site 11 | -0.1 | 0.08 | -1.68 | 0.094 | -0.1 | 0.08 | -1.68 | 0.094 |
| Site 12 | 0.3 | 0.08 | 4.12 | < 0.001 | 0.3 | 0.08 | 4.12 | < 0.001 |
| Site 13 | 0.5 | 0.08 | 7.12 | < 0.001 | 0.5 | 0.08 | 7.12 | < 0.001 |
| Site 14 | 1.2 | 0.08 | 16.00 | < 0.001 | 1.2 | 0.08 | 16.00 | < 0.001 |
| Site 15 | 0.5 | 0.08 | 7.19 | < 0.001 | 0.5 | 0.08 | 7.19 | < 0.001 |
| Site 16 | -0.1 | 0.08 | -1.81 | 0.071 | -0.1 | 0.08 | -1.81 | 0.071 |
| Site 17 | 0.1 | 0.08 | 1.62 | 0.105 | 0.1 | 0.08 | 1.62 | 0.105 |
| Site 18 | 0.6 | 0.08 | 7.54 | < 0.001 | 0.6 | 0.08 | 7.54 | < 0.001 |
| Site 19 | -0.2 | 0.08 | -2.32 | 0.020 | -0.2 | 0.08 | -2.32 | 0.020 |

**Table E.** Revenue per day from all categories: full models

|  | Reference: Baseline | | | | Reference: Availability | | | |
| --- | --- | --- | --- | --- | --- | --- | --- | --- |
|  | Estimate | SE | *t* value | *p* | Estimate | SE | *t* value | *p* |
| Modelling of the mean (identity link): |  |  |  |  |  |  |  |  |
| Intercept | 602.3 | 5.78 | 104.21 | < 0.001 | 586.7 | 7.26 | 80.79 | < 0.001 |
| Temperature (°C) | 0.4 | 0.28 | 1.42 | 0.157 | 0.4 | 0.28 | 1.42 | 0.157 |
| Time (Days; Baseline) | 2.4 | 6.02 | 0.41 | 0.685 | 2.4 | 6.02 | 0.41 | 0.685 |
| Time (Days; Availability) | -19.5 | 6.24 | -3.12 | 0.002 | -19.5 | 6.24 | -3.12 | 0.002 |
| Time (Days; Availability plus Size) | 35.1 | 6.93 | 5.07 | < 0.001 | 35.1 | 6.93 | 5.07 | < 0.001 |
| Baseline | *reference* | *reference* | *reference* | *reference* | 15.6 | 5.20 | 3.00 | 0.003 |
| Availability | -15.6 | 5.20 | -3.00 | 0.003 | *reference* | *reference* | *reference* | *reference* |
| Availability plus Size | -34.1 | 5.04 | -6.76 | < 0.001 | -18.5 | 5.19 | -3.56 | < 0.001 |
|  |  |  |  |  |  |  |  |  |
| Modelling of the variance (log link): |  |  |  |  |  |  |  |  |
| Intercept | 4.3 | 0.05 | 79.26 | < 0.001 | 4.3 | 0.05 | 79.26 | < 0.001 |
| Site 2 | 0.2 | 0.08 | 2.77 | 0.006 | 0.2 | 0.08 | 2.77 | 0.006 |
| Site 3 | 0.6 | 0.08 | 7.86 | < 0.001 | 0.6 | 0.08 | 7.86 | < 0.001 |
| Site 4 | -0.7 | 0.08 | -9.07 | < 0.001 | -0.7 | 0.08 | -9.07 | < 0.001 |
| Site 5 | 0.0 | 0.08 | -0.02 | 0.983 | 0.0 | 0.08 | -0.02 | 0.983 |
| Site 6 | 0.1 | 0.08 | 0.94 | 0.346 | 0.1 | 0.08 | 0.94 | 0.346 |
| Site 7 | 0.3 | 0.08 | 3.43 | 0.001 | 0.3 | 0.08 | 3.43 | 0.001 |
| Site 8 | 0.7 | 0.08 | 8.96 | < 0.001 | 0.7 | 0.08 | 8.96 | < 0.001 |
| Site 9 | -0.8 | 0.08 | -10.60 | < 0.001 | -0.8 | 0.08 | -10.60 | < 0.001 |
| Site 10 | 1.3 | 0.08 | 16.50 | < 0.001 | 1.3 | 0.08 | 16.50 | < 0.001 |
| Site 11 | -0.7 | 0.08 | -8.97 | < 0.001 | -0.7 | 0.08 | -8.97 | < 0.001 |
| Site 12 | -0.4 | 0.08 | -4.74 | < 0.001 | -0.4 | 0.08 | -4.74 | < 0.001 |
| Site 13 | 0.9 | 0.08 | 12.28 | < 0.001 | 0.9 | 0.08 | 12.28 | < 0.001 |
| Site 14 | 0.6 | 0.08 | 7.44 | < 0.001 | 0.6 | 0.08 | 7.44 | < 0.001 |
| Site 15 | 0.5 | 0.08 | 7.03 | < 0.001 | 0.5 | 0.08 | 7.03 | < 0.001 |
| Site 16 | -0.9 | 0.08 | -11.49 | < 0.001 | -0.9 | 0.08 | -11.49 | < 0.001 |
| Site 17 | 0.0 | 0.08 | 0.00 | 0.998 | 0.0 | 0.08 | 0.00 | 0.998 |
| Site 18 | 0.8 | 0.08 | 10.53 | < 0.001 | 0.8 | 0.08 | 10.53 | < 0.001 |
| Site 19 | -0.6 | 0.08 | -7.43 | < 0.001 | -0.6 | 0.08 | -7.43 | < 0.001 |

**Replication**

The current study is a replication and extension of two pilot studies (Hollands et al., 2018; Pechey et al., 2019) both of which used six sites. We pre-specified in the analysis plan that we would test replication of Pechey et al, and our criteria for determining replication were that 1) the direction of effects is consistent and 2) a significant one-sided test of equivalence (TOST) for a difference of ±3% (Δ*_L_* = −3%, Δ*_U_* = 3%). We also decided after data collection to conduct an exploratory replication test for Hollands et al using the same criteria.

Pechey et al (2019) reported a drop in energy purchased of -6.9% (-11.7% to -1.7%) (Availability *vs* baseline); the current study showed a drop in energy purchased of -4.8% (-7.0% to -2.7%) (Availability *vs* baseline). The two one-sided tests (TOST) analysis showed that the results of the current study and those of the pilot were not statistically equivalent, *t*(23) = 0.81, *p* = 0.215, suggesting that there was no evidence for replication based on the ±3% equivalence boundary. However, equivalence was achieved at the slightly larger threshold of 3.2%, *t*(23) = -1.725, *p* = 0.050.

Hollands et al (2018) showed a drop in energy of -8.9% (-16.7% to -0.4%) (Size vs baseline). The current study showed a drop in energy purchased of -6.6% (-7.9% to -5.4%) (Availability plus Size vs Availability). The TOST analysis showed that the results of this study and the pilot were statistically equivalent, *t*(23) = -2.42, *p* = 0.012 (Δ*_L_* = −3%, Δ*_U_* = 3%).

## Sensitivity analyses

We conducted a sensitivity analyses whereby the number of main meals and pie of the day meals sold were manually adjusted based on incorrect button pressing that we identified while on site visits. These adjusted data provided similar results to the main results, suggesting that the findings are robust to these errors in till button pressing (see Table F)

**Table F.** A comparison of the primary analysis and sensitivity analysis for the primary outcome

|  | Primary analysis | | | Sensitivity analysis | | |
| --- | --- | --- | --- | --- | --- | --- |
|  | Estimate | Confidence intervals | *p* | Estimate | Confidence intervals | *p* |
| Availability vs Baseline | -4.8% | -7.0% to -2.7% | < .001 | -7.0% | -9.4% to -4.7% | < .001 |
| Availability + Size vs baseline | -11.5% | -13.7% to -9.3% | < .001 | -11.4% | -14.3% to 8.4% | < .001 |
| Availability plus Size vs Availability | -6.6% | -7.9% to -5.4% | < .001 | -4.4% | -5.0% to -3.9% | < .001 |

**Moderation analyses**

We calculated a series of correlations between measures of intervention implementation and model estimates of the intervention effectiveness in each site (see Table G). It is unclear from these analyses, which were underpowered, whether the implementation of either intervention across all intervention categories was linked with intervention effectiveness, with effect sizes in the predicted direction for the Size intervention, and in the opposite direction for the Availability intervention. Furthermore, none of the correlations were significant at a Bonferroni adjusted *α* = .0125.

When focussing on the implementations in the main meal category, for which we have the most complete data, and which account for the largest proportion of energy purchased across all categories, there is evidence that better implementation is linked with greater reductions in energy purchased for one of the two Availability outcomes but none of the Size outcomes (see Table G).

**Table G.** Table of correlations between intervention implementation and effectiveness

|  | Availability effectiveness | | Availability effectiveness (corrected) | | Size effectiveness | | Size effectiveness (corrected) | |
| --- | --- | --- | --- | --- | --- | --- | --- | --- |
|  | *Rho* | *p* | *Rho* | *p* | *Rho* | *p* | *Rho* | *p* |
| Availability implementation (overall) | 0.34 | .154 | 0.06 | 0.804 | - | - | - | - |
| Availability implementation (main meals) | -0.50 | .029 | -0.61 | 0.005* | - | - | - | - |
| Size implementation (overall) | - | - | - | - | -0.28 | 0.241 | -0.18 | .463 |
| Size implementation (main meals) | - | - | - | - | 0.01 | 0.971 | 0.12 | 0.61 |

*Note. ** significant at Bonferroni adjusted α = .0125. Positive correlation coefficients indicate that the effectiveness decreases with better implementation. Negative coefficients indicate that the effectiveness increases with better implementation. Availability effectiveness: Availability vs baseline site level effects for the primary outcome. Availability effectiveness(corrected): Availability effectiveness Availability vs baseline site level effects for the corrected primary outcome. Size effectiveness: Availability plus Size vs Availability site level effects for the primary outcome. Size effectiveness(corrected): Availability plus Size vs Availability site level effects for the corrected primary outcome. Availability implementation (overall): the percentage point change in less healthy products on sale between baseline and Availability. Availability implementation (main meals): the percentage point change in less healthy products on sale between baseline and Availability. Size implementation (overall) = the number of products reduced in size * the magnitude of the reductions in size. Size implementation (main meals) = the number of main meals reduced in size * the magnitude of the reductions in size.
