# Supplementary material for: Impact of decreasing the proportion of higher energy foods and reducing portion sizes on food purchased in worksite cafeterias: A stepped-wedge randomised controlled trial
Source: PLoS Med. 2021 Sep 14;18(9):e1003743. doi: 10.1371/journal.pmed.1003743 (PMC8439477; doi:10.1371/journal.pmed.1003743)
Supplement: Attachment — Submitted filename: Response to reviewers PLOS v3.1.docx [file pmed.1003743.s005.docx]

We would like to thank the editor and three reviewers for providing such detailed comments. We have gone through and responded to each comment and made changes to manuscript where we judged it to be necessary.

**Requests from the editors:**

**To your data statement, please add details for a non-author contact for readers who may be interested in inquiring about access to the study data.**

*Response:* The company that collaborated with us wished to remain anonymous and shared their data with us on condition that we do not share it beyond the research team. By providing contact details we would de-anonymise the company.

**Please avoid "energy purchased" in the title, perhaps with a different form of words.**

*Response:* We have changed the title to read “food purchased”. We hope that this improves the clarity of the title.

OLD: Impact of decreasing the proportion of higher energy foods and reducing portion sizes on energy purchased in worksite cafeterias: a stepped-wedge randomised controlled trial

NEW: Impact of decreasing the proportion of higher energy foods and reducing portion sizes on food purchased in worksite cafeterias: a stepped-wedge randomised controlled trial

**Please combine the "Methods" and "Findings" subsections of your abstract. Please add a new final sentence to the combined subsection, which should begin "Study limitations include ..." or similar and quote 2-3 of the study's main limitations.**

*Response:* We have made these changes and added in two key limitations to the abstract, p.2:

“Study limitations include using energy purchased as the primary outcome as it was not possible to objectively measure energy consumed. Furthermore, the research team were provided with transaction-level sales data from each site. Participant-level data were not available.”

**Please quote study dates in the abstract.**

*Response:* We have added this into the abstract, p.2:

“and lasted for 25 weeks (May – November 2019)”

**Please mention the unit of randomization in the abstract; and list the relevant secondary outcomes.**

*Response:* We have added these details into the abstract, p.2:

“19 cafeterias were randomised to the order in which they introduced the two interventions.”

“The secondary outcomes were energy (kcal) purchased from non-interventions categories per day, total energy purchased per day, and revenue.”

**After the abstract, we will need to ask you to add a new and accessible "Author summary" section in non-identical prose. You may find it helpful to consult one or two recent research papers published in PLOS Medicine to get a sense of the preferred style.**

*Response:* We have written an author summary on p.3 of the manuscript:

### “Why was this study done?

### Overconsumption of energy from food and drink contributes to the high and rising rates of obesity in many countries

- Creating healthier food environments is key to enabling healthier diets
- There is insufficient real-world evidence on the best ways to improve food environments

### What did the researchers do and find?

- Two interventions were evaluated: *i*. Availability: replacing some higher energy foods with lower energy foods, *ii*. Size: reducing the portion size of some higher energy foods
- A randomised stepped-wedge trial in 19 cafeterias spanning 25 weeks was conducted
- The Availability intervention reduced energy purchased by 4.8% and the Availability plus Size intervention reduced energy purchased by 11.5%

### What do these findings mean?

- Replacing some higher energy foods in cafeterias with lower energy options and reducing the portion size of some higher energy foods are both effective strategies for reducing energy purchased in worksite cafeterias
- These interventions can contribute to broader strategies to reduce energy intake out of the home, as part of national and international efforts to tackle overweight and obesity.”

**Could "written consent", mentioned in the Methods section, be described as "informed"?**

*Response:* We have made this change, p.5

“The research team obtained informed consent…”

**Please rename figure 1 "Study site flowchart" or similar.**

*Response:* We have made this change, p.6

“Figure 1. Study site flowchart”

**Please add an additional sentence or two to the first paragraph of your Discussion, so as to summarize the study and its findings.**

*Response:* We added in a new opening sentence to the discussion to summarise the study, p.20. The following sentences already summarised the findings, so we have not edited these.

*“*The current study evaluated two interventions that altered the food environments of 19 worksite cafeterias located throughout England, Scotland, and Wales.“

**Please avoid claims such as the "largest" or "first", as in the Discussion section, and where needed add "to our knowledge" or similar.**

*Response:* We added “to our knowledge” to the first of these claims in the introduction on p.5:

“The current study – the largest of its kind, to our knowledge – aimed… ”

We also removed this claim from the discussion on p.19

**Throughout the text, please adapt reference call-outs to the following style: "... food and drinks [4,5].".**

*Response:* We have made this change

**Please remove the information on data sharing, funding and competing interests from the end of the main text. In the event of publication, this information will appear in the article metadata via entries in the submission form.**

*Response:* We have made this change

**Please remove all iterations of "[Internet]" from your reference list.**

*Response:* We have made this change

**Noting reference 11, please ensure that all references include full access details.**

*Response:* We have gone through the references and made sure all details are now there.

**Please rename the attached CONSORT checklist "S1_CONSORT_Checklist" or similar, and refer to it by this label in your Methods section. Please adapt the checklist so that individual items are referred to by section (e.g., "Methods") and paragraph number rather than by line or page numbers, as the latter generally change in the event of publication.**

*Response:* We have changed the name as requested, references this checklist in the methods, and altered the items to refer to section and paragraph number.

Comments from the reviewers:

*** Reviewer #1:

**The authors test large-scale interventions decreasing availability of 'higher energy' products. There sample consisted especially of male staff working in manual occupation labor force. Their results show a decrease of higher energy foods sold in cafeterias when the proportion of these higher energy foods was decreased and when portion sizes were reduced. The paper is well written and gives a clear overview of how the interventions have been implemented and the results of implementing these interventions. I have only minor comments/questions.**

***	During intervention period no recipes could be altered or meals could be changed, while this was the case prior to the study. What happened when an ingredient fell short during the intervention period? Was the ingredient replaced? Was the product taken away from the assortment?**

*Response:* There were only a few occasions when a product could not be sourced or there was another reason a meal needed to be changed (e.g., a special event/themed meals). In such events we were sent the energy content of the new product that was sold that day and we substituted that new value into the analysis for the days in which it occurred. We have added this to the methods on p.7:

“There were seven recorded instances of a meal deviating from the planned meal on that day. When this occurred the new meal’s energy content was used and not that of the planned meal.”

The far more common occurrence was that they had a surplus of a specific ingredient, and unfortunately (due to the strict rules throughout the trial) this led to food waste.

***	Was there a logbook with notes on problems for example, when one ingredient fell short, when the till didn't work?**

*Response:* Problems were monitored and recorded in spreadsheets. As stated above, the far more common problem was left over food, leading to food waste. While this was a problem for the sites, it did not impact the study results or conclusions.

***	How were the dished placed? Were 'more healthy' products placed between the other products or placed at a specific place (for example, offered at the beginning of the counter?). Placement of dishes everywhere the same? Refer to research on where dishes are placed affect purchases.**

*Response:* The guidelines and training for catering staff highlighted that the only changes should be the interventions and they should not change anything else to influence purchasing (such as changing placement, advertising certain products, etc). They were also instructed that the new products should replace the location of the ones that they replaced, where possible. As such, during the availability intervention, healthier products are likely to have been placed where less-healthy ones were previously. We have added a new section to the discussion to emphasise this, p.22:

“The Availability intervention involved replacing some higher energy products with lower energy ones. While this is conceptually distinguished from an intervention in which the placement of items is systematically altered, the act of removing and replacing products may also change the placement of the products on sale – with sites asked to place healthier products where less-healthy ones were previously. While changing the order of products may affect food selection and purchasing [12], it is unclear whether changes to placement necessitated in the Availability intervention could have contributed to the effect observed, particularly given variation in site layout and displays usually featuring a mix of the healthier vs. less-healthy options within the study categories investigated.”

***	When taking away the "more energy" products in the availability intervention, did you replace the products highest in energy?**

*Response:* We removed some of the higher energy products. However, this was a negotiation between the research team and the catering providers. We have added these details to the supplement for readers further interested in these decisions:

“The decision regarding which products to remove was a negotiation between the catering providers (who wanted to keep certain high selling products) and the research team (who wanted to remove the highest energy products). We were successful in making the case for replacing the highest energy main meals (i.e. pizza). It did not prove possible to replace the highest energy side dish (i.e. chips). It was, however, possible to reduce the portion size of the chips and other higher energy sides such as roast potatoes and wedges.”

***	During the availability intervention the aim was to have about 50% high energy of most type of meals (e.g. cooked main meals) while for snacks this was 75 or 80%. Why? Are there not enough lower energy snacks?**

*Response:* These targets were set following discussion and negotiation with the three catering providers and the supermarket group. As you identify, the main barrier was the ability for the catering providers to procure lower energy snacks. There was also some resistance from the catering providers who thought that their customers would not be interested in buying lower energy snacks. We have added a sentence to the manuscript to highlight the rationale for these targets, p.8:

“The planned degree of implementation – such as reducing higher energy sweet snacks from 90% to 80% – was negotiated with the catering providers and was primarily determined by their ability to procure lower energy options.”

***	Were there control cafeterias in which no interventions were implemented so that you could control for larger fluctuations in meal choice based on for example public policy campaings, news issues, seasonal fluctuations?**

*Response:* There were no separate control cafeterias as the reviewer describes, as in a stepped wedge design (SWD) the cafeterias act as their own controls. Using such a design means that certain types of temporal bias can be minimised (e.g. if there had been a national policy campaign affecting all sites on one date). Furthermore, the incorporation of time effects in models helps control for the temporal trends in these data.

***	Was the reduction in size observable by the participants? The authors argue that there probably were no compensatory effects. Do you have individual tickets to look at whether more side dishes were chosen or more desserts when the size and/or availability intervention was implemented?**

*Response:* The reduction in size was on average 14% for several targeted products. We didn’t measure systematically whether the customers detected the difference, however from speaking to the catering staff there were very few comments from the customers.

It would in theory be possible to explore if more side dishes or desserts were selected based on the data we have. But there are many different possible ways in which compensation effects may occur and testing all the different possible food choices that may increase as a possible compensation effect would be post-hoc exploration and would increase the likelihood of type I errors. The key message here is that - on average - energy purchased decreased. While some participants may have bought an extra chocolate bar (or similar) during the intervention periods, the net effect was a reduction in energy purchased.

We do not argue that our data suggests no compensation effects, but rather that our data suggest that net energy purchased decreases – even if the composition of a meal may have changed. We also cite well-controlled laboratory and real-world experiments that specifically test the compensatory effect hypothesis – and these find no evidence that it occurs. However, to provide further clarity on this point we have altered the wording in the discussion, p.22:

**“**It is unknown if compensation effects did occur in our study as we did not test for this, however the net effect of the interventions was a reduction in energy purchased.”

***	Will health posters as such already affect behavior (intentions? Accessibility health?) were these posters displayed during the baseline period?**

*Response:* These posters stated that a new health initiative was being trialled however the specific interventions were not described. There was also contact information so that the customers could voice any complaints/comments to the supermarket group to inform their healthy eating policies after the study was completed. These were put up during the baseline period and kept throughout the trial, and therefore are unable to explain the drop in energy purchased when Availability was implemented, or the further drop in energy purchased with Size was added to Availability.

***	Participants do not have to put the food on the plate themselves (i.e. choosing how many spoons they take)? So the meals were served to them?**

*Response:* All main meals and sides were served to the customers by catering staff. This allowed us to control the portion size of the different products. We have edited the text to make this clear on p.12:

“The portion sizes of items that were served using ladles – such as for stew, or scoops – such as for chips – were reduced by either instructing staff to use fewer ladles or scoops when serving customers”

***	Can you control for out of stock of higher energy meals? If one wants to choose a high energy meal but it is out of stock, one probably chooses a low energy meal. Were the high energy meals often out of stock or chosen first? This could mean that people still had the intention to eat high energy meals but were restricted by the fact that the product was out-of-stock (which could decrease the chances of building a "habit" of choosing less high energy products).**

*Response:* Unfortunately, we do not have data for whether the meals ran out of stock during service. While this possibly occurred some of time, this was not detected as an issue during any of our site visits.

***	Are participants aware of the energy content of the dishes, is this labeled on the products?**

*Response:* The product labels only contained the product name, the price, and whether it contained any allergens.

***	Did participants complain about a change in assortment or sizes?**

*Response:* There appeared to be a fear amongst the catering managers that there would be lots of complaints, but once the study was underway very few were brought to our attention. We have added this point to the discussion, p.22:

“There were further concerns by catering staff that the changes would not be acceptable to customers. However, once the study started very few comments or complaints were brought to our attention.”

***	There were more transactions in the availability and availability + size condition versus the baseline. Can you find out whether these were unique customers or customers that bought a meal and then later on went back to buy another meal?**

*Response:* Unfortunately not. We requested access to unique identifiers however these data were not shared with us due to the company’s policies about data privacy. Therefore we do not know the extent to which different transactions represent different customers. We have added this to the discussion on p.22:

“The company that provided the sales data were unable to provide individual-level data due their data privacy rules. We therefore do not know the extent to which different transactions represented different customers.”

***	Could the fact that people also buy less high energy products in the non-intervention categories indicate that you created a more healthy mindset by for example the poster or by choosing or seeing more 'less energy products' in the intervention categories?**

*Response:* That is one possible interpretation, and we do mention that this is possible on p.21, line 17-19. However, as we discuss in this section and the results, there were some issues with data quality for this outcome and therefore we have tried to make it clear that readers should not over interpret this finding, and it would need replicating before further conclusions can be reached.

***	One of the supplementary materials was password protected so could not be opened "excel document "availability characteristics by site")**

*Response:* Apologies for this error. We have re-uploaded this document and it should be possible to view now

***** Reviewer #2:**

**This is a statistical review of manuscript PMEDICINE-D-20-06163R1 reporting the results of a stepped-wedge randomised trial assessing the impact of decreasing the proportion of higher energy foods and reducing portion sizes on energy purchased in worksite cafeterias. Overall, I found the manuscript clear and easy to follow and only have minor comments listed below.**

***	Abstract: Please add the number of cafeterias (19) and number of groups/wedges (10) in the methods paragraph.**

*Response:* We have added this into the abstract, p.2:

“19 cafeterias were randomised in 10 groups to the order in which they implemented the two interventions.”

***	I find Figure 1 difficult to understand, partially due to potential confusion between steps and groups/wedges. To make it clearer, I would suggest relabelling the boxes "Availability" and "Availability+Size" as Availability (Step 1)" and "Availability+Size (Step 2)" respectively and, in the 20 boxes, simply indicate the week when the corresponding intervention was started e.g. "Week 5" and "Week 13" for Group 1.**

***Response:*** The Availability and Availability plus Size interventions are not the “steps” and therefore we have not changed the labels to represent (Step 1) as suggested. In this design, the individual sites which introduce the interventions are the steps – see Figure 2 with new labels to make this clearer.

We decided not to add the dates to this figure as this would duplicate information from Figure 2 and make the figure too crowded. The purpose of Figure 1 is to show how each site moved through the study rather than to illustrate the study design (see Figure 2 for this).

***	Figure 2 is a great way to visualise the overall design.**

*Response:* Thank you for this comment.

***	In the sample size calculation section, please indicate the corresponding design (number of steps and number of groups) as well as the intra cluster correlation assumed.**

*Response:*

The sample size was based on pragmatic considerations (i.e. the number of suitable sites available) as there were no *a priori* estimates of effect size and ICC on which to base such a calculation. The sample size statement given in the paper therefore instead provides a simpler statement of what effect size might be detected by a basic t-test given a sample size of n=19. Other more efficient designs such as an adaptive design with sample size re-estimation were considered, but a timely stop/go decision was not possible to implement in this scenario.

***	The primary outcome is the total energy (kcal) sold by a cafeteria per day which does not take into account potential variations in the number of "clients". The list of covariates includes number of transactions per day as a proxy for the number of customers; however, the methods do not appear to describe covariate adjustments. Please add a description of the full models and included covariates in the methods. Instead of adding the daily number of transactions as a covariate in the model,** **I wonder whether it might be useful to model the average energy per transaction (i.e. the ratio of total daily energy divided by daily number of transactions) as a separate outcome.**

*Response:*

The methods section does report the model covariates (p.13 lines 19-27), further details can be found in the Analyses sub-section of the methods, and the full model tables in the supplement from S2 onwards.

We also considered modelling the ratio of total energy and transactions, but it is not simple model to build or interpret for the ratio of two variables. Sometimes there were no transactions (days on which a site was closed). These are two reasons we have not used this approach.

***	Please clarify the distribution and link function used in the model e.g. normal distribution with identity link.**

*Response:* This detail has been added to the Analyses section on p.14:

“The identity link function (i.e. normal distribution) was used for modelling the mean and the sigma link function (i.e. log10) was used for modelling the variance.”

***	I note that the effect of the intervention is reported as a relative difference (%) together with its confidence interval instead of a mean difference in energy. Please clarify how these quantities were derived from the models. Were log transformations used?**

*Response:* The models were run using energy purchased per day as the key outcome, therefore the model outputs for the two interventions are expressed in terms of mean difference in energy (See Table S2 in the supplement). We converted this to % change using the daily mean energy purchased during baseline, and the estimated change during Availability and Availability + Size. We chose to do this conversion as it is more easily interpreted given the different sizes of sites and is the typical format for presentation of these data in previous research.

***	A 2-sided 95% confidence interval is reported for the primary outcome (see top of Page 15); however, the power calculation mentions a one-sided test with 5% which would correspond to a 2-sided 90% confidence interval (5% on each side). Please clarify/correct.**

*Response:*

The sample size statement was illustrative only of what effect size might be observed under strong assumptions of unknown accuracy, given a sample size of n=19 and having no *a priori* estimates available. As such, the pre-registered statistical analysis plan also never planned for a 1-sided t-test to be used but instead use a regression analysis approach.

***	When reporting the results, including tables and figures, please indicate that the outcome is the energy purchased daily.**

*Response:* We have reworded the Figure 3 title in the main manuscript to make it the outcome clearer, p.18 (see below). We have also added “per day” to each of the titles in the supplemental tables to make this clearer.

“Figure 3. The effects of Availability and Availability plus Size on energy (kcals) purchased per day relative to baseline.”

***	In Table S2, please clarify what the estimates represent including the exact units. Please also clarify units related to covariates. For example, does the estimate of 36.77 for temperature indicate that for each unit increase in temperature (one degree Celsius?), the total daily energy purchase increases by 36.77 kcal?**

*Response:* Correct, daily temperature was in degrees Celsius and that is the correct interpretation. We have added this unit to the temperature variable in each of the supplemental tables. We have also added the unit of time to these tables: days.

***	The full model includes a linear time trend for each period (baseline, availability, availability plus size). These effects assume a linear trend with day as the unit of time. Has the assumption of linearity been checked? One alternative could be to model time trends based on the study week (1 to 25) instead of day and without assuming a specific relationship (e.g. linear). This could be done by adding week as a class/categorical effect across the entire study period. Has this been considered?**

*Response:* We also considered the suggested analysis however the weekly time effect differed by site and there was only one observation per week per site, therefore such a model would have been over-specified.

Before starting the analysis we conducted many data checks including plotting the data for each site and overall. The visual checks of the data were consistent with no trend or approximately linear trends. Also, a key feature was that mean and variability at each site changed in a regular way by day of the week, and we were keen to include this term in the model.

***	Please consider reporting at most one decimal when reporting beta coefficients for absolute changes in kcal (cf Table S2 for example).**

*Response:* We have made this change in the supplemental tables S2-S5.

***	Please consider including a plot of the average daily energy purchased over time with the day within each period on the x-axis.**

*Response:*

We have modelled the total daily energy purchased. Sites are of different sizes, and “step” onto the intervention at different times and therefore the time spent pre-intervention and post-intervention is different at each site. The suggested plot would be misleading as there would random be jumps upwards along the plot and then later random jumps downwards, reflecting the timing of the step a site was randomised to. For this reason we prefer not to present such a plot.

***	In place of the individual cafeteria estimates presented in the supplement, please consider using visual representation showing daily or weekly estimates over time by cafeteria.**

*Response:*

We prefer to retain the current overall estimates as a succinct summary in the body of the paper. However in response to this suggestion we have added the much longer 19-page “Intervention cals by site over time - with interv1+2 times marked.pdf” into the supplementary material, showing the suggested information.

***	Please indicate whether missing data was present and, if so, what methods were applied.**

*Response:* One form of missing data was the lack of energy content estimates for 2% of the products on sale. We imputed the energy content of these remaining products that were missing energy information by using the energy content from another catering provider (there were 3 in the study) if the product description was the same. After this process, there were 7 remaining individual products that we could not get energy content for. These final 7 were imputed by taking the mean energy content of 3 products with the same name sold at major UK supermarkets. We describe this process on p.13, line 5-8.

***	According to Table 3, the energy purchased per day increased during the availability period compared to the baseline period; however, the results from the models suggest the opposite direction. I understand that the modelled estimates are subject to adjustments (e.g. due to temporal trends or random effects); however, some explanation including potential plots could help understand this apparent discrepancy.**

*Response:* We agree, without context this may appear confusing. We have added a note to highlight this, p.17

*“Note.* The unadjusted energy purchased is marginally higher in the Availability period relative to baseline – in contrast to the modelled effect. This is primarily due to the increased number of transactions that took place during the Availability period”

***	Given than Table 3 shows raw/unadjusted estimates, I would suggest presenting it before presenting the results from the models.**

*Response:* We have made this change

***	From my perspective, the primary outcome appears to be more a measure of intervention fidelity rather than a measure of impact (e.g. weight loss, change in diet, etc.). I would suggest adding this as a limitation.**

*Response:* The outcomes of interest are changes in diet and subsequently the health benefits that are associated with these changes, as the reviewer states. However, to get objective measurements of these variables on such a large scale would not be possible. We instead use this objective measure of food purchased as a close approximation of food consumed. We discuss the limitations of this approach in the discussion on p.22, line 7-11. “Intervention fidelity” is technically defined as whether the intervention was implemented as planned (eg <https://bmjopen.bmj.com/content/3/11/e003555>), and we address this in several sections throughout the paper. Most notably Table 2, where we report that on average that 76% of the Availability targets were met and 83% of the Size targets were met.

The two interventions that we tested would not necessarily translate to behaviour change in terms of purchasing less energy, although we hypothesised that it would. It’s possible (and a frequent concern) that people may simply adjust their purchasing habits in the presence of these interventions to ensure they still obtain at least the same amount of energy in their diet (for example, by buying an extra portion of chips if the portion size is reduced). Whereas we show that on average purchasing behaviour changes, and therefore – we assume – a person’s consumption of energy changes.

-Laurent Billot

*** Reviewer #3:

Thank you for the opportunity to review this manuscript. The present paper examines the effect of changing the availability and portion sizes of higher energy foods in 19 worksite cafeterias used by 20,327 employees on the energy purchased. The study is very impressive in terms of its methodological rigorousness, sample size and it is addressing a timely topic. My comments follow:

**The introduction should flesh out more the conceptual and theoretical framework. The TIPPME framework is only briefly mentioned in the methods section. However, the classification of "availability x product" and "size x product" is very hard to understand without additional information. It seems likely that not all readers are familiar with the TIPPME typology and why and how this might be different from nudging.**

*Response:* We agree and have fleshed out this section of the introduction to make this clearer, p.4:

“There are many interventions that alter such food environments and a recent Typology of Interventions in Proximal Physical Micro-Environments (TIPPME) [11] describes six of these interventions, each of which can be applied to products (e.g., the food itself), related objects (e.g., the table on which the food is served or consumed), or the wider environment (e.g., the cafeteria’s structure or layout). The interventions described in this typology overlap with the more general concept of “nudging”, but the typology aims to provide more precisely operationalised classifications of different intervention types.”

**Please briefly elaborate the reasons for and implications of the implemented intervention sequence in the introduction and discussion. The "availability" intervention was introduced first followed by the combination "availability & portion size reduction". Why was a "availability" intervention singled out but not a "portion size" intervention?**

*Response:* We originally hoped to test both interventions in isolation and then in combination, but did not have access to the number of sites to be sufficiently powered for this. We had to select one to test in isolation, and opted for availability. This was done primarily for two reasons: *i.* having conducted pilot studies for each of these interventions, the results were most promising for Availability and we privileged testing of what appeared to be the most promising intervention based on evidence at that time, *ii.* it made more sense to reduce portion sizes after changing products, as opposed reducing portion sizes first, and then removing products. We have added this justification into the method, p.9-10:

“While ideally both Availability and Size would have been investigated individually as well as together, due to power constraints we needed to select one. We opted for Availability for two reasons: *i.* the pilot study favoured the Availability intervention and we wanted to test the more promising intervention in isolation, *ii.* it made more sense to reduce portion sizes after changing products, as opposed to reducing portion sizes first, and then removing products.”

**How are the 20 steps in Figure 1 related to the 25 week numbers in Figure 2? In order to harmonize Figure 1 and 2, (if possible), I suggest to include the weekly periods instead of steps in Figure 1.**

*Response:* The 20 steps in Figure 1 are equivalent to the “visual steps” in Figure 2. We have kept Figure 1 the same, however we have updated Figure 2 to make the link clearer:

**Figure 2.** Stepped-wedge study design

**The results show only for 3 out of 19 cafeterias a significant "availability" effect (site 10, 14, 15) and for 6 out of 19 cafeterias a significant "availability & portion size reduction" effect (site 10, 14, 15 and 4, 6, 9). Why were these sites more successful? I suggest to elaborate more on this important aspect in discussion. Are they belonging to the same catering company? Is it related to the size of the assortment or cafeteria?**

*Response:* There were many factors that differed between the cafeterias including catering provider, degree of implementation, region of the country, demographic characteristics of customers, size of the site, and size of the cafeteria. Furthermore, data from an individual site would not provide the power to necessarily detect an effect – especially with a conservative adjustment for multiple testing such as the Bonferroni method that we used. This could mean that the intervention did change behaviour at more cafeterias, we just were unable to detect the effect in those individual cafeterias due to greater variation in the data or a lack of power. Due these issues, it is not possible to know which characteristics are important for an effect to be detected. However, we agree that this is an important question and so we have expanded on our discussion of this question, p.21:

“It should be noted, however, that there was variation in outcomes between the different cafeterias. We observed statistically significant effects in only three of the 19 cafeterias for Availability and six of the 19 cafeterias for Availability plus Size. This is due partly to the conservative adjustment that was used for multiple testing. It can also be explained by the limited power that a single site has for detecting a change in energy purchased. The extent to which the interventions were implemented, which varied across cafeterias, will also have contributed to different effect sizes. Moderation analyses reported in supplementary materials provide mixed support for this latter interpretation. However, the power to test for any moderation effects was very limited. The cafeterias differed in many other ways including the region of the country in which they were based and the demographic characteristics of the employees. These and other unknown factors may also have contributed to variations in effects across sites.”

**There are some sites which show an increase in energy purchased (e.g. site 5, 11). Are there any observations why this (counterintuitive) effect occurred?**

*Response:* While it is true that these were numerical increases in energy purchased relative to baseline, when adjusting for covariates (e.g. number of transactions) none of these effects were statistically significant based on our prespecified criteria and therefore it would be inappropriate to overinterpret these increases. However, we did conduct several checks to determine if the sites with increases were those that had poorer intervention fidelity. One of these sites (17) had the poorest intervention implementation with a 50% success rate. However, two others (5 & 7) had adequate implementation (75% & 74%) for availability. While this does not mean that it was not a contributing factor, there was no statistically clear indication in our data of an important relationship.

**Concerning the secondary outcomes, "unexplained outliers and erratic time trends" were observed. What are potential explanations for this? Is there a relation to the primary outcomes? I suggest to elaborate this aspect more in the discussion.**

*Response:* Prior to running the main analyses we ran a number of checks including plotting the data by outcome. The primary outcome did not have these trends and had minimal outliers. One of the secondary outcomes – specifically, energy purchased in non-intervention categories - had many outliers and the amount of energy that was purchased appeared to increase and decrease at different points throughout the trial, seemingly as much in the pre-intervention period as the intervention period. The site managers were unable to offer any explanation for this. Next, we analysed the data by food category. For example, the non-intervention categories outcome included breakfasts, sandwiches, and “other”, which were rarer items that did not fall into one of the main categories. We examined each of these categories and found that all three categories were contributing to the outliers and time trends. These unusual patterns are common in real world data and unfortunately in this scenario we were unable to detect what was underlying the outliers and trends. We have elaborated on this in the discussion as the reviewer recommends, p.21:

“Thorough examination of the data and discussions with site managers did not detect the cause of these data characteristics. However, the same checks were conducted for all other outcomes and were not found to influence the primary outcome or the revenue outcome.”

**p. 18, line 18; it is stated that there were "large" interventions effects which ranged between a -4.8% to -11.5% reduction in energy purchased, relative to the baseline. What is the metric for classifying these as a "large" effect? Conversely, on p. 19, line 15; it is stated that the interventions resulted in a "small" drop in revenue. However, the drop was between -2,6% to 5,7%.**

*Response:*

We agree with the reviewer. Upon reflection we could not come up with an appropriate method of classifying the effect size into such simplistic categories and therefore we have removed these terms.

**The discussion is partly repeating the introduction and the results (e.g., p. 18, line 23ff).**

*Response:* Agreed – it is repeating the introduction, however we think this is an important point that merits highlighting, so we have reworded this to be less repetitive, p.20:

“Those in manual occupations have – on average - worse health outcomes and higher body mass indexes (BMIs) compared to those in non-manual occupations [7] and many interventions that aim to improve healthy eating only exacerbate existing inequalities [20]. The effectiveness of the Availability and Size intervention in the current sample therefore indicates the potential to improve health equitably, assuming that the interventions are not more effective among those in managerial or higher socio-economic-status groups.”
