# Supplementary material for: Impact of decreasing the proportion of higher energy foods and reducing portion sizes on food purchased in worksite cafeterias: A stepped-wedge randomised controlled trial
Source: PLoS Med. 2021 Sep 14;18(9):e1003743. doi: 10.1371/journal.pmed.1003743 (PMC8439477; doi:10.1371/journal.pmed.1003743)
Supplement: Attachment — Submitted filename: Response to reviewers S3.docx [file pmed.1003743.s006.docx]

**Noting PLOS' data policy,** [**https://journals.plos.org/plosmedicine/s/data-availability**](https://journals.plos.org/plosmedicine/s/data-availability)**, our firm view is that a solution needs to be found which provides a route for interested readers to access study data that does not involve contacting the article authors. We suggest that making an anonymized dataset available along with the paper or in a publicly accessible repository is the best way to achieve this, but are happy to discuss the matter further as needed.**

*Response:* Following our email correspondence with the Editor (Richard Turner), we propose the following wording to be added to the data availability statement:

*“This study includes sales data from the collaborating supermarket, which were received weekly from May 2019 to November 2019. Due to contractual restrictions to the use of the sales data, these data cannot be made openly available.  Any requests to access the data can be directed to our administrators (*[*bcbd.administrator@medschl.cam.ac.uk*](mailto:bcbd.administrator@medschl.cam.ac.uk)*). The collaborating supermarket wished to remain anonymous and any requests for further use of these data must be sent via our administrators who will pass the request to the collaborating supermarket.”*

**Please adapt the final sentence of the "Methods and findings" subsection of the abstract so that this discusses only study limitations.**

*Response:* The final sentence of the abstract was a study limitation – the lack of participant level data impedes our ability to draw causal inferences about the intervention effects. We have edited the final two sentences to make this clearer (p2, line 24):

“Study limitations include using energy purchased as the primary outcome (and not energy consumed), and availability only of transaction-level sales data per site (and not individual-level data).”

**Please remove the statement about ethics and consent from the end of the main text, as this should be in the Methods section if not already present there.**

*Response:* We have removed this. This ethics information was already in the methods section (p.5, lines 10-13)

**Please use the general style "... week 5" throughout the text, although numbers should be spelt out at the start of sentences.**

*Response:* We have made three edits accordingly, all on p.9, line 18.

**Please adapt reference call-outs so that there are no spaces within the square brackets (e.g., "...drinks [4,5].").**

*Response:* We have edited the citation delimiter accordingly.

**Please remove footnotes (short explanations can perhaps be included in the text).**

*Response:* The two footnotes on p.13 were removed and the content added to the text.

**Please adapt reference citations so that 6 author names are listed, followed where appropriate by "et al.".**

*Response:* We have made this change

**Please add "U S A" to reference 38, and ensure that journal names are abbreviated consistently.**

*Response:* We have made this change

**Comments from the reviewers:**

***** Reviewer #2:**

**I thank the authors for addressing my comments. My only remaining comment relates to the power calculation.**

**I understand the pragmatic nature of the sample and the fact that the number of cafeterias was more or less fixed; however, one should still report the effect size detectable by the design for a certain level of power. Given this is a cluster (stepped-wedge) design, one should also take the ICC in consideration. If the ICC is unknown, it would be interesting to know the effect size detectable under reasonable assumptions about the ICC. From my perspective, it would also make sense for the power calculation to be redone assuming a 2-sided test of 5% to match the analysis planned and conducted.**

*Response RE ICC:* Unfortunately the data we have is at the aggregate transaction level at each site, and not at participant level. It is therefore not possible to calculate an ICC for between participant variability. We have clarified this in the abstract (see previous comment).

*Response RE power:* In terms of an a priori sample size statement, an indicative statement based on a simple t-test was given even though the planned analysis was always pre-planned to be a repeated-measures mixed effects model using 2-sided tests. As participant-level data was not available, an a priori cluster sample size calculation is not appropriate for the current trial design. Regarding using a 1-sided or 2-sided test-based power calculation, this is of course a highly arguable point. However this statement had no influence on i) the actual sample size recruited, which was based primarily on pragmatic considerations, or ii) the analysis method employed. Purely for reference, a 2-sided t-test might have 70% power to detect an effect of 6.5%, or a 2-sided t-test might be able to detect an effect size of 7.3% with 80% power.

*For reference, current sample size statement (paper p5):*

*“*The sample size calculation was based on data from two similar studies [14, 15]. We conservatively used the largest estimate of SD (0.111) when analysis was on the log scale. A one-sided t-test at 80% power with 5% significance level would require *n* = 19 cafeterias to detect an effect size of 6.5% or greater reduction in energy purchased. We selected a one-sided test, reflecting existing evidence [14, 15] [12, 13]*”*
