# Supplementary material for: Impact of decreasing the proportion of higher energy foods and reducing portion sizes on food purchased in worksite cafeterias: A stepped-wedge randomised controlled trial
Source: PLoS Med. 2021 Sep 14;18(9):e1003743. doi: 10.1371/journal.pmed.1003743 (PMC8439477; doi:10.1371/journal.pmed.1003743)
Supplement: Attachment — Submitted filename: Response to reviewers S4.docx [file pmed.1003743.s007.docx]

We are pleased to hear that the paper will be accepted following these minor changes. As mentioned in an email to [plos@medicine.org](mailto:plos@medicine.org) we would like to negotiate the publication date to be fixed to a particular day. This is so that we can coordinate our university’s press release with the publication of the paper.

Due to several members of the team having annual leave, we request that publication be delayed to **14^th^ September 2021**. Please contact either of the two corresponding authors to discuss this if you have any questions.

Our responses to the editors’ comments:

**To the segment of the title preceding the colon, please add "... in a UK supermarket" or similar.**

*Response:* The worksite cafeterias were based in the distribution centres of a major UK supermarket group, which would make the full title:

Impact of decreasing the proportion of higher energy foods and reducing portion sizes on food purchased in worksite cafeterias based in the distribution centres of a UK supermarket group: a stepped-wedge randomised controlled trial

We agree with the editor that titles should convey necessary context of research, however we believe that “worksite cafeterias” is sufficient detail and that any further information makes the title excessively long and harder to read. This further information can be found in the abstract and therefore we would prefer not to add it to the title.

**In the author summary, please use the active voice in at least one point, e.g., "We carried out a randomised ...".**

*Response****:*** We have made this change (p.3, line 10):

- **“**We carried out a randomised stepped-wedge trial in 19 cafeterias spanning 25 weeks”

**In the penultimate point, please soften the wording, e.g., "Replacing some higher energy foods ... both appear to be effective strategies for reducing ...".**

*Response****:*** We have made this change (p3, Line 17):

- “Replacing some higher energy foods in cafeterias with lower energy options and reducing the portion size of some higher energy foods both appear to be effective strategies for reducing energy purchased in worksite cafeterias”

**The current final sentence of the Introduction is really an element of discussion, and we ask you to either move it to the Discussion section or combine it with the preceding sentence (e.g., "So as to estimate the individual and combined effectiveness of availability and size interventions, we designed ...").**

*Response****:*** We have reframed this sentence as an aim, to make it more appropriate to belong in this section (p5, line 3):

“We also aimed to investigate the impact of implementing an availability intervention singly, as well as in combination with a size intervention, to estimate the individual and combined effectiveness.”

**In the Methods section, was that "written informed consent"?**

*Response****:*** That is correct, we have added that word in (p5, line 12):

“The research team obtained written informed consent from a representative of the supermarket group on behalf of the participating sites.” **Numbers can be used at some additional points in the text, e.g., "... 3 of the 19 cafeterias ..." at the end of p.19.**

*Response****:*** We have made these changes and searched the document for further instances (p.19, line 28):

“We observed statistically significant effects in only 3 of the 19 cafeterias for Availability and 6 of the 19 cafeterias”

And p.9, line 17 and 25:

“The baseline period lasted between 4 and 13 weeks”

**“**The Availability plus Size intervention period, therefore, lasted 4 to 13 weeks”

**Can the attached CONSORT checklist be adapted to remove "Page no" at the top of the rightmost column?**

***Response:*** Unfortunately not, this is the template the authors of the CONSORT extension created for this purpose and that part of the PDF is not editable
